# Australian *Allograpta* Osten Sacken (Diptera, Syrphidae)

**DOI:** 10.3897/zookeys.513.9671

**Published:** 2015-07-15

**Authors:** Ximo Mengual, F. Christian Thompson

**Affiliations:** 1 Zoologisches Forschungsmuseum Alexander Koenig, Leibniz-Institut für Biodiversität der Tiere, Adenauerallee 160, D–53113 Bonn, Germany; 2 Department of Entomology, National Museum of Natural History, Smithsonian Institution, Washington, D.C., USA

**Keywords:** Australia, flower fly, hoverfly, new species, description, identification key

## Abstract

*Allograpta terraenovae* **sp. n.** and *Allograpta notiale* **sp. n.** are described from Australia. Notes on the Australian species of *Allograpta* and an identification key to them are also given. The lectotype of *Allograpta javana* Wiedemann is designated, and the species *Syrphus pallidus* Bigot is synonymized under *Allograpta australensis* (Schiner).

## Introduction

*Allograpta* Osten Sacken, 1875 (Diptera, Syrphidae) is a world-wide genus with its greatest diversity in the Neotropics (Thompson et al. 2010). Adult flies are pollinators and flower visitors and larvae are mostly predators of soft-bodied Hemiptera (Rojo et al. 2003), although secondarily some species are phytophagous (Nishida et al. 2003, Zuijen and Nishida 2011, Weng and Rotheray 2009). The genus was recently reviewed (Mengual et al. 2009) and based on morphological characters and molecular evidence (Mengual et al. 2008a, 2008b, 2012), six different genera are currently recognized instead of the previous subgenera (Thompson 2012).

Only three *Allograpta* species were known for the Australian fauna, *Allograpta alamacula* Carver, 2003, *Allograpta australensis* (Schiner, 1868) and *Allograpta pallida* (Bigot, 1884) (Mengual et al. 2009), but no key or review of these has been published. The aim of the present work is to review the Australian species of this flower fly genus and to describe two new species.

## Material and methods

New species are described in full, following the terminology by Thompson (1999). The holding collection of each specimen is indicated between square brackets after the label information. The abbreviations used for collections follow the standard of the *Systema Dipterorum* (Thompson 2013), and their equivalents are given below:

ANIC Australian National Insect Collection, CSIRO, Canberra City, Australia.

AMS Australian Museum, Sydney, Australia.

CNC Canadian National Collections of Insects, Ottawa, Canada.

NMW Naturhistorisches Museum Wien, Vienna, Austria.

QM Queensland Museum, South Brisbane, Australia.

USNM National Museum of Natural History, Washington D.C., United States of America.

UMO University Museum of Natural History, Oxford, United Kingdom.

ZMUC Zoological Museum, University of Copenhagen, Copenhagen, Denmark.

ZFMK Zoologisches Forschungsmuseum Alexander Koenig, Bonn, Germany.

Italics in the description of type labels denote handwriting, the contents of each label is enclosed within double quotation (“ ”), and the individual lines of data are separated by a double forward slash ( // ). All measurements are in mm and were taken using a reticule in a Leica M165 C microscope (Wetzlar, Hesse, Germany). Photographs were composed using the software Zerene Stacker 1.04 (Richland, Washington, USA) based on images of pinned specimens taken with a Canon EOS 7D mounted on a P–51 Cam-Lift (Dun Inc., Virginia, USA) and the help of Adobe Lightroom (version 5.6) (San Jose, California, USA). Distribution maps were created using SimpleMappr (Shorthouse 2010).

### Key to the Australian species of *Allograpta*

**Table:** 

1	Metasternum bare; wing with apical brown macula. Male genitalia large, visible from dorsal view (Fig. 1)	***alamacula* Carver**
–	Metasternum pilose; wing without apical macula. Male genitalia smaller, usually underneath tergum 5^th^ (Figs 2, 3, 5)	**2**
2	Katepimeron, coxae, pro- and mesotarsus yellow (Fig. 3)	***australiensis* (Schiner)**
–	Katepimeron and coxae black; pro- and mesotarsus with apical tarsomeres dark brown to black (Figs 5, 7, 15, 16)	**3**
3	Postalar callus yellow pilose (Figs 5, 7, 9, 11); occiput yellow pilose (Figs 5–8); male frontal triangle yellow pilose (Figs 5, 6), with small medial brown macula dorsad to antenna (Fig. 6)	***terraenovae* sp. n.**
–	Postalar callus partially black pilose (Figs 10, 12, 16); occiput black pilose on dorsal 1/3 (Figs 10, 13–16); male frontal triangle black pilose (Fig. 13), with large brown macula dorsad to antennae and reaching laterally around antenna (Fig. 13)	***notiale* sp. n.**

**Figures 1–8.**
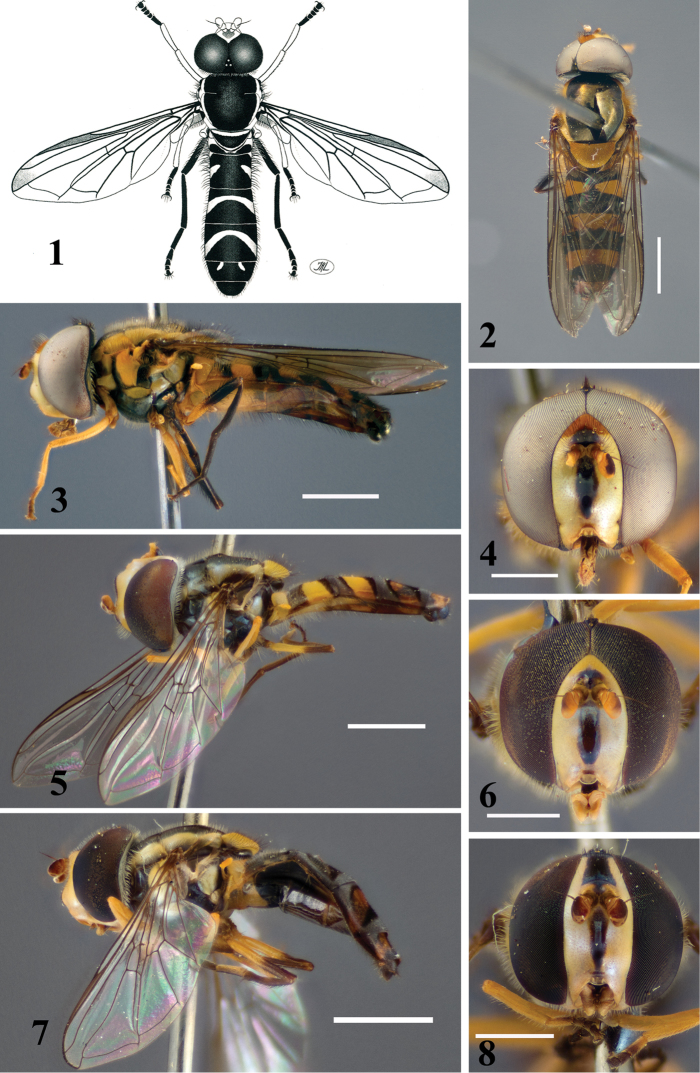
Australian *Allograpta* species: **1** *Allograpta alamacula*, male, dorsal **2–4** *Allograpta australensis*: **2** male, dorsal **3** male, lateral **4** male, frontal **5–8** *Allograpta terraenovae* sp. n.: **5** male (holotype), lateral **6** male (holotype), frontal **7** female (paratype), lateral **8** female (paratype), frontal. Scale for lateral and dorsal views: 2 mm. Scale for frontal views: 1 mm.

**Figures 9–16.**
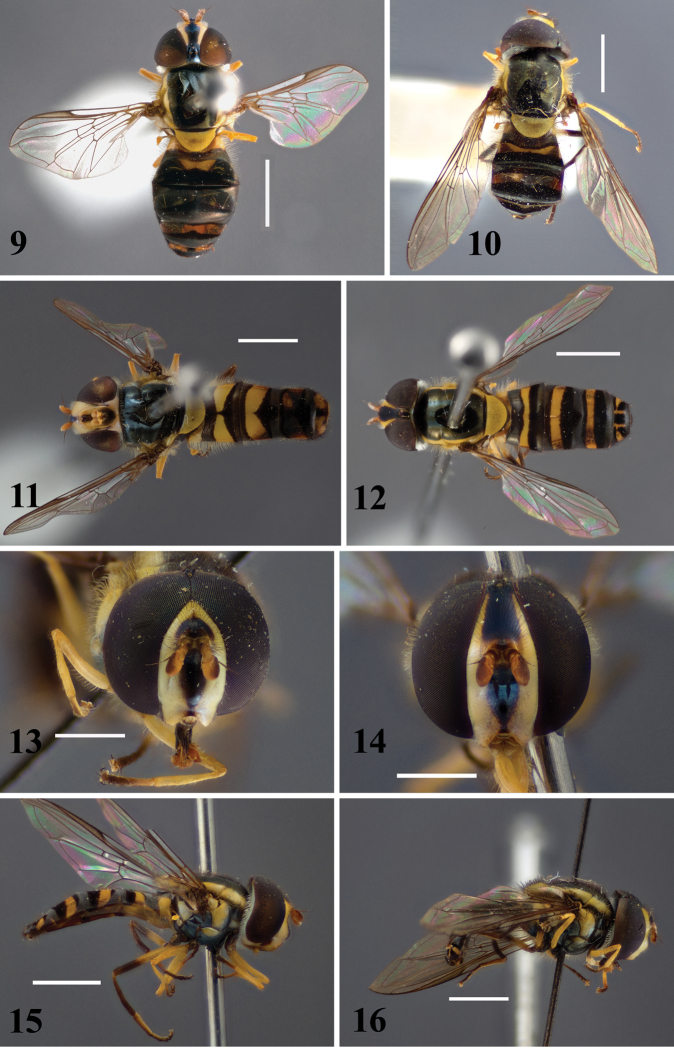
Australian *Allograpta* species: **9** *Allograpta terraenovae* sp. n., female (paratype), dorsal **10** *Allograpta notiale* sp. n., male (holotype), dorsal **11** *Allograpta terraenovae* sp. n., male (holotype), dorsal **12–16** *Allograpta notiale* sp. n.: **12** female (paratype), dorsal **13** male (holotype), frontal **14** female (paratype), frontal **15** female (paratype), lateral **16** male (holotype), lateral. Scale for lateral and dorsal views: 2 mm. Scale for frontal views: 1 mm.

## Australian species of *Allograpta*

### Allograpta alamacula

Taxon classificationAnimaliaDipteraSyrphidae

Carver, 2003

Figure 1 , 17

Allograpta alamacula Carver in Carver and Thompson 2003: 37; fig. 1 (habitus), fig. 2 (male genitalia). Type-locality: Australia, Queensland, Indooroopilly [HT male, ANIC].

#### Diagnosis.

Face straight, with large tubercle; oral opening about 2 times as long as wide, with oral apex at level of antennal base; antennal pits confluent; plumula absent; subscutellar pile fringe absent; wing broadly bare basomedially, with apical dark macula; alula broad, as broad as cell bm; metasternum bare; abdomen elongate.

#### Biology.

Carver reared her species from maggots preying on the whitefly species *Aleurocanthus t-signatus* (Maskell, 1896) (Hemiptera, Aleyrodidae) (Carver and Thompson 2003).

#### Distribution.

**Figures 17–18.**
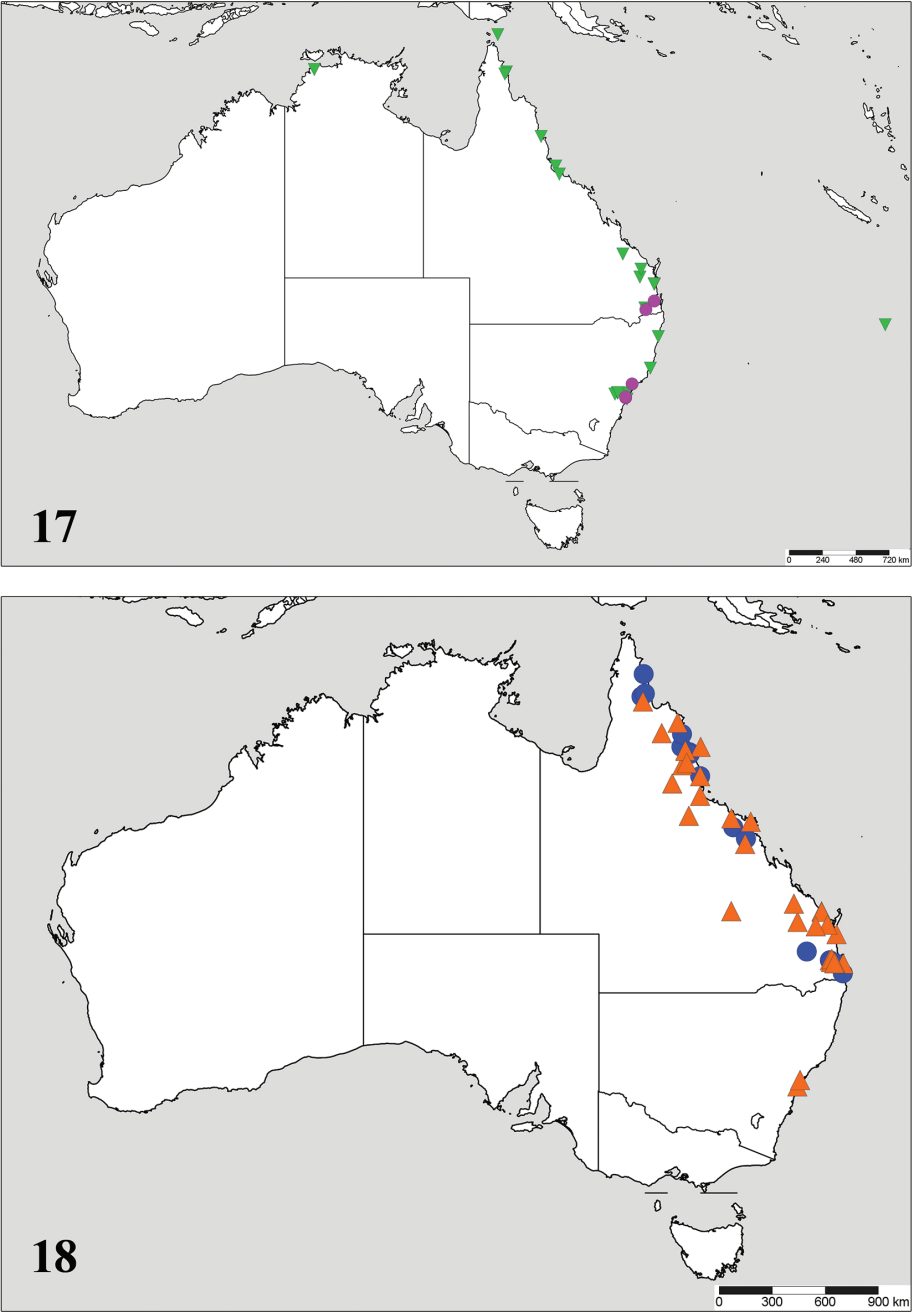
Distribution of the Australian *Allograpta* species: **17** *Allograpta alamacula* (pink circles) and *Allograpta australensis* (green inverted triangles) **18** *Allograpta terraenovae* sp. n. (orange triangles) and *Allograpta notiale* (blue circles).

Australia (New South Wales, Queensland); Fig. 17.

### Allograpta australensis

Taxon classificationAnimaliaDipteraSyrphidae

(Schiner, 1868)

Figures 2–4 , 17

Melithreptus australensis Schiner, 1868: 347. Type-locality: Australia, Sydney [HT female, NMW]. Holotype presumably lost (Vockeroth 1971: 1628), corroborated by first author (XM).
Syrphus pallidus Bigot, 1884: 93. Type-locality: “Australie” [HT male, UMO]. Kertész 1910: 124 (cat. cit.); Hull 1936: 194 (distr.). **Syn. n.**
Sphaerophoria australensis : Kertész 1910: 135 (cat. cit.); Curran and Bryan 1926: 129 (descr. note, distr.); Hull 1936: 195 (distr.).
Allograpta javana (Wiedemann, 1824) of Australian authors; Hardy, 1933: 13 (as *Sphaerophoria javana*; distr., syn. of *australensis* Schiner).
Allograpta australensis : Vockeroth 1971: 1628 (rev. status, diff. *iavanus* Wiedemann, figures); Thompson and Vockeroth 1989: 441 (cat. cit.).
Allograpta pallida : Thompson and Vockeroth 1989: 442 (cat. cit.).

#### Diagnosis

(modified from Vockeroth 1971). Face produced forward below, ventral margin of head long and oblique; yellow maculae of 2^nd^ tergum (sometimes fused in female) narrowed laterally but extending narrowly to margins of tergum; yellow fasciae of 3^rd^ and 4^th^ terga strongly narrowed laterally and not extending to margins of terga; apex of metafemur and broad base and apex of metatibia brown to yellow-brown or black, the yellow annulus (ring) at middle of metatibia very poorly defined (some males without annulus).

#### Distribution.

Australia (Queensland, New South Wales, Northern Territory, Norfolk Island); Fig. 17.

#### Biology.

In the ANIC collection, there is a series of males and females collected as larvae on flowers of *Eucalyptus*.

#### Remarks.

The holotype of *Syrphus pallidus* Bigot was examined and was found to be the same species as *australensis* Schiner.

### Allograpta javana

Taxon classificationAnimaliaDipteraSyrphidae

(Wiedemann, 1824)

Syrphus iavanus Wiedemann, 1824: 34. Type-locality: Indonesia, Java [LT here designated, NMW].
Syrphus javanus : Wiedemann 1830: 131 (emendation, redescription); Zimsen 1954: 19 (no type specimen in Copenhagen), here verified.
Sphaerophoria javana : Kertész 1910: 136 (cat. cit.).
Xanthogramma javana : Bezzi, 1928: 73 (descr. (A, P), distr.); Hull 1936: 195, 1937: 83 (distr.).
Miogramma javana : Frey 1946: 165 (comb.).
Helenomyia javana : Bańkowska 1962: 311 (comb.).
Allograpta javana : Vockeroth 1969: 130 (comb.).
Allograpta iavana : Thompson and Vockeroth 1989: 441 (cat. cit.).

#### Diagnosis

(modified from Vockeroth 1971). Face nearly vertical, ventral margin of head shorter and more nearly horizontal; yellow maculae of 2^nd^ tergum (usually fused in female) and yellow fasciae of 3^rd^ and 4^th^ terga not narrowed laterally, extending to margins of terga in their full width; apex of metafemur and broad base and apex of metatibia dark brown to black, the yellow annulus at middle of metatibia sharply defined.

#### Distribution.

India, Sri Lanka, north to Mongolia, China, Korea, Primorye (Primorsky Krai, Russia) and Japan, east to New Guinea, Solomon Islands and Fiji.

#### Remarks.

*Allograpta javana* (Wiedemann) was confused with *Allograpta australensis* (Schiner) until Vockeroth (1971) separated the two. All material we have examined is of *Allograpta australensis* and undescribed species, so the status of the *Allograpta javana* in Australia is still dubious (Vockeroth also did not know *Allograpta javana* from Australia). *Allograpta javana* is similar to *Allograpta australiensis* in that the katepimeron is yellow, but differs from *Allograpta australiensis* in the vertical face, not projecting anteriorly (Vockeroth 1971: 1629, figures 1 and 2); abdominal fasciae not narrowed laterally and extending to margins in their fullest widest and the yellow medial annulus on metatibia distinct. Other morphological characters to distinguish these two species are: anepimeron black in *Allograpta javana*, including dorsomedial portion of anepimeron (dorsomedial anepimeron yellow in *Allograpta australensis*); male frons yellow pilose in *Allograpta javana* (mainly black pilose with some yellow pili in *Allograpta australensis*); male with occiput yellow pilose in *Allograpta javana* (male with occiput black pilose on dorsal 1/3 and yellow pilose on basal 2/3 in *Allograpta australensis*); and usually metabasitarsomere pale in *Allograpta javana* (metabasitarsomere dark in *Allograpta australensis*).

*Allograpta javana* is a species complex, whose components probably should be recognized as full species. In at least eastern New Guinea and throughout Oceania, the face is entirely yellow [*Allograpta distincta* (Kertész, 1899: 177)], whereas the western component always has a broad black medial vitta (typic form). In Oceania, *Allograpta amphotera* Bezzi (1928: 74) and *Allograpta nigripilosa* Hull (1944: 52) are related more closely to *Allograpta javana* (typic form) than to *Allograpta distincta*, as they retain the black facial vitta.

Zimsen (1954: 19) mentions that there was no type specimen in the Zoological Museum of Copenhagen (ZMUC) or in Vienna (NMW), but Groll (2013) indicates that some Diptera and Hymenoptera of the Wiedemann collection went to Vienna via W. von Winthem. At NMW, there is a pinned male labelled: “*Java* // Coll. Winthem” “*javannus* // det. Wiedem.” “*javanus Wid* // *Java*” “*LECTOTYPE* // *Allograpta* // *iavana* // *WIEDEMANN* // K. Ghorpade de*s.* 19*83*” [red] “LECTOTYPE // *Allograpta* // *javana Wied.* // de*s.* X. Mengual 20*14*” [red]. We do agree with Ghorpadé in considering this specimen as part of the type series studied by Wiedemann. This specimen is here designated as the lectotype to fix and ensure the universal and consistent interpretation of the name.

### Allograpta terraenovae

Taxon classificationAnimaliaDipteraSyrphidae

Thompson sp. n.

http://zoobank.org/FAA81060-E319-4773-8F12-D8D9EA4D3BAB

Figures 5–8 , 9 , 11 , 18

Allograpta 88–13 Thompson in *litt*.

#### Type locality.

AUSTRALIA: Queensland, Jowalbinna, 6.7 km west of, 15°45'S, 144°12'E.

#### Types.

*Holotype* male labelled: “6.7 km W ‘Jowalbinna’ // H.S., Qld 15°45'S, 144°12'E // 11 May 1989 // G. and A. Daniels” “Australian Museum // K402264” “*Holotype* // *Allograpta* // *nsp. 88*–*13* // *Thompson*” [red] “HOLOTYPE // *Allograpta* // *terraenovae* // *Thompson 2014*” [red] [AMS].

*Paratypes*: AUSTRALIA, NEW SOUTH WALES: Marsfield, vii.1976, C.E. Chadwick [1♀ AMS; AMK 404830]; Mooney Mooney Creek near Gosford, 3.xii.1976, D.K. McAlpine [1♀ USNM; AMK 410638]. QUEENSLAND: 6.7 km West of ‘Jowalbinna’ H.S., 15°45'S, 144°12'E, 11.v.1989, G. & A. Daniels [1♂ AMS; AMK 402265]; Brisbane, 7.ix.1927, J. Mann [1♀ QM; UQIC 220474]; Brisbane, C. Deane [1♀ QM; UQIC 220475]; Brisbane, 10.x.1916, H. Hacker [1♀ QM; UQIC 222111]; Brisbane, C.F. Ashby [2♂ 2♀ ANIC; ANIC 33153]; Bundaberg, viii.1971, H. Frauca [1♂ ANIC; ANIC 33157]; 2 miles North of Bundaberg, 26.vi.1971, Tea-tree swamp, H. Frauca [1♀ ANIC; ANIC 33159]; 14 km West by North Hope Vale Mission, 4.v.1981, D.H. Colless [1♀ ANIC; ANIC 33160]; 20 miles South of Ingham, 1.ix.1956, C. Deane [2♀ QM; UQIC 220476, 220477]; 3km S Mt Spurgeon, 1100 m., 20–22.xi.1997, C. Burwell, open forest [1♀ QM, UQIC 221457]; 7–14 miles West of Herberton, via Watsonville, 1.v.1967, D.H. Colless [1♀ ANIC; ANIC 33154]; Brisbane, 25.x.1953, F.M. Hull [2♂ CNC]; Flinders Mem. Park, 23.v.1968, J.W. Boyes [1♂ CNC; USNM ENT00249235]; Brisbane, Toowong, 26.v.1968, J.W. Boyes [1♀ CNC]; Bluff Range, near Biggenden, ca. 2750 ft., v.1971, H. Frauca [1♀ ANIC]; Atherton, 3–18.xi.1972, A.M. Hemmingsen [1♂ ZMUC]; Carnarvon Stn, nr Piebald Spring (CN1M1), 821 m., 13.xii.2010–15.vi.2011, C. Zwick & C. Wilson, malaise trap, *Eucalyptus*/*Callistemon* in rocky gully [1♀ QM; UQIC 222109]; Dunk Island, 25.viii.1927 [2♀ QM; UQIC 222110, 220478]; Great Sandy National Park, Cooloola Section, 1–5.x.1996, Winterton, D.K. Yeates, C. Lambkin, malaise trap [1♀ QM; UQIC 220473]; Mandalay Point, Great Barrier Reef, 13.viii.1986, De Beer [1♂ QM; UQIC 220479]; Mount Glorious, 6.xi.1965, C.F. Ashby [1♂ 1♀ ZFMK, 1♂ ANIC, 1♂ USNM; ANIC 33152, USNMENT 01028878]; Mount Glorious, 8.xi.1965, J.K. Guyomar [2♂ ANIC; ANIC 33156]; Mount Glorious Scrub Creek Road, Brisbane Forest Park, 17–24.x.1997, N. Power, malaise trap [1♂ QM; UQIC 220471]; Mt. Abbott, upper slopes, 700–900 m., 10–12.iv.1997, C. Burwell [2♂ 5♀ QM; UQIC 221451, 221452, 221447, 221448, 221450, 221454, 221456; 2♀ ZFMK; UQIC 221449, 221453]; Mt. Robert, 5km SW, 300 m., 23.x.2000, S. Wright, brigalow [1♀ QM; UQIC 222112]; Petrie Park, Mayborough, 15.xi.1993, G. & A. Daniels [1♀ AMS; AMK 402262]; Samsonvale Cemetary, 9.vi.1996, S.G. Evans [1♀ QM; UQIC 221462]; Scrub Road, Brisbane Forest Park, 12–19.Ix.1997, S. Winterton, N. Power, D. White, malaise trap [1♀ QM; UQIC 220472]; The Blunder, 20.ix.1969, C.F. Ashby [1♀ ANIC; ANIC 33158]; Toomba, Site 1, 390 m., 14–15.xii.2006, S. Wright, rainforest/paddock edge [1♂ QM; UQIC 222113]; Undara Volcanic National Park, The Bluff, 19.vii.1998, J.& R. Skevington [2♂ QM; UQIC 220459, 220461; 1♂ ZFMK, UQIC 220460]; Undara Volcanic National Park, The Bluff, 770 m., 11.vi.1997, J.& R. Skevington, hilltop [6♂ QM; UQIC 220463, 220464, 220465, 220466, 220467, 220468, 220470; 1♂ ZFMK, UQIC 220462]; Hilltop ~21 km South of Coen, 14.137538°S, 143.240945°E, 945 m., 2.xii.2014, J.H., A.M. & A.W. Skevington [26♂ CNC; CNC373686, CNC373687, CNC373688, CNC373689, CNC373690, CNC373691, CNC373692, CNC373693, CNC373694, CNC373695, CNC373696, CNC373697, CNC373698, CNC373699, CNC373700, CNC373701, CNC373702, CNC373703, CNC373704, CNC373705, CNC373706, CNC373707, CNC373708, CNC373709, CNC373710, CNC373711]; Kroombit Tops National Park, hilltop, 24.44818°S, 150.93520°E, 21.xii.2014, J.H., A.M. & A.W. Skevington [4♂ CNC; CNC384420, CNC384422, CNC384423, CNC384424]; Kroombit Tops National Park, hilltop, 24.448183°S, 150.935200°E, 22.xii.2014, J.H., A.M. & A.W. Skevington [1♂ CNC; CNC384468]; Sheoak Ridge Nature Reserve near Julatten, 16.645258°S, 145.403366°E, 7.xii.2014, J.H., A.M. & A.W. Skevington [3ex CNC; CNC371573, CNC371574, CNC371575]; Sheoak Ridge Nature Reserve near Julatten, Summit of hilltop in dry sclerophyll forest, 16.645258°S, 145.403367°E, 8.xii.2014, J.H. Skevington [34♂ CNC, 4♂ ZFMK, 4♂ USNM; CNC371588, CNC371589, CNC371590, CNC371591, CNC371592, CNC371593, CNC371594, CNC371595, CNC371596, CNC371597, CNC371598, CNC371599, CNC371600, CNC371601, CNC371602, CNC371603, CNC371604, CNC371605, CNC371606, CNC371607, CNC371608, CNC371609, CNC371610, CNC371611, CNC371612, CNC371613, CNC371614, CNC371615, CNC371616, CNC371617, CNC371618, CNC371619, CNC371620, CNC371621, CNC371622, CNC371623, CNC371624, CNC371625, CNC371626, CNC371627, CNC371628, CNC371629]; University of Queensland, St. Lucia, 16.viii.1994, G. Gordh, pupated 17.viii.1994, emerged 22.viii.1994 [1♂ QM; UQIC 220480]; Wilston, Brisbane, 28.vi.1998, S.G. Evans, pupated 2-3.vii.1998, emerged 10.vii.1998 [2♀ QM; UQIC 221460, 221461]; Wilston, Brisbane, 22.vi.1998, C.J. Burwell, on *Eucalyptus* feeding on lerps [1♀ QM; UQIC 221463]; Eidsvold, xii.1922 [1♂ ANIC; ANIC 33155]; Kuranda, F. P. Dodd [1♂ USNM; USNMENT 01028922]; 52km SWbyS of Mt. Garnet, 700 m., 18.05°S, 144.52°E, 28.v.1977, I.F.B. Common & E.D. Edwards [1♀ USNM; USNMENT 01028948].

#### Diagnosis.

Species with metasternum pilose and katepimeron and coxae black. Very similar to *Allograpta notiale* sp. n., but *Allograpta terraenovae* sp. n. has yellow pilosity on postalar callus and occiput, and male frons is also yellow pilose, with a small medial brown macula dorsad to antennae.

#### Description.

Male. *Head*. Face yellow, with medial black vitta, shiny, yellow pilose; gena yellow on anterior 1/2, black posteriorly, yellow pilose; lunule yellow laterally, black medially; frontal triangle yellow except narrowly black immediately dorsad to lunule, shiny, yellow pilose; vertical triangle black, shiny, black pilose; occiput mostly black, yellow on ventral 1/9, white pilose on basal 2/3, and yellow pilose on dorsal 1/3; antenna orange, except basoflagellomere brownish black on dorsal 1/3, black pilose; arista brownish orange.

*Thorax*. Postpronotum yellow, shiny; propleuron black, sparsely silvery-white pollinose, white pilose; scutum black except broad yellow laterally, sparsely black pollinose, yellow pilose; postalar callus yellow, yellow pilose; scutellum yellow, black pilose; plumula orange; calypter light brownish except medial 1/3 of margin and fringe yellow; pleuron black except anepisternum yellow on posterior 2/3, katepisternum yellow on dorsal 1/3, and katatergum yellow on dorsal 4/5, sparsely silvery-white pollinose, white pilose; metasternum pilose. *Legs*: coxae black, sparsely silvery-white pollinose, white pilose; trochanters brownish black; pro- and mesofemora yellow, white pilose except black pilose on apical 1/3 dorsoposteriorly; metafemur yellow on basal 2/3, black apically, pale pilose basally, black pilose apically; pro- and mesotibiae yellow, yellow pilose; metatibia black, black pilose; tarsi brownish black, black pilose. *Wing*: hyaline, bare on basal 2/3, microtrichose apically; microtrichose on apical 1/4 of cell r_1_, apical 1/2 cell r_2+3_, apical 2/3 of cell r_4+5_, apical 3/4 of cell dm, apical 1/2 of cell cup, and broadly along posterior margins of alula and anal lobe.

*Abdomen*. 1^st^ tergum yellow except narrowly black on apical margin, yellow pilose; 2^nd^ tergum black except for large yellow medial fascia, which may be narrowly separated medially, shiny along basal and apical margins, black pollinose bordering yellow fascia, yellow pilose on basal 3/4, black pilose apically; 3^rd^ and 4^th^ terga black except for large arcuate yellow fascia, shiny along basal and apical margins, black pollinose bordering yellow fascia, black pilose; 5^th^ tergum black, except for large triangular yellow maculae, black pilose; sterna yellow, white pilose except 4^th^ sternum black pilose. *Male genitalia* black, shiny, black pilose.

*Female*. Similar to male except for normal sexual dimorphism and as follows: frons yellow laterally (about 1/4 of frons width) with a medial, broad, black vitta (about 1/2 of frons width); abdominal fascia narrower than in male, very narrow medially on terga 3 and 4 looking like two joined maculae.

#### Distribution.

Australia (New South Wales, Queensland); Fig. 18.

#### Etymology.

The specific epithet is derived from the combination of *terra* (land, earth) and *nova* (new), and it refers to Australia. Species epithet to be treated as an adjective.

#### Biology.

There is a female with a puparium (AMK 404830) that was collected as a larva preying on *Eucalyptolyma maideni* Froggatt, 1901 (Hemiptera, Psyllidae). Another female (UQIC 221463) was reared from a larva found feeding on lerps (Hemiptera, Psyllidae) on *Eucalyptus*.

### Allograpta notiale

Taxon classificationAnimaliaDipteraSyrphidae

Thompson sp. n.

http://zoobank.org/0AB09328-B716-4F48-8FDF-83CF3BB56F36

Figures 10 , 12–16 , 18

Allogratpa 88–14 Thompson in *litt*.

#### Type locality.

AUSTRALIA: Queensland, Collinsville, 20°33'S, 147°50'E.

#### Types.

*Holotype* male labelled: “Collinsville, Q. // 15–9–1950 // E.F.Riek.” “Australian // National // Insect // Collection” [green] “*Allograpta* // *88*–*14* // Det. X. Mengual, 201*2*” “ANIC Database No // 29 033161” “HOLOTYPE // *Allograpta* // *notiale* // *Thompson 2014*” [red] [ANIC].

*Paratypes*: AUSTRALIA, QUEENSLAND: Brisbane, 25.x.1953, F.M. Hull [1♂ CNC]; 18 miles North of Cairns, 13.v.1970, R. & J. Matthews [1♂ ZFMK, 1♂ ANIC; ANIC 33162]; 2 miles West of Kuranda, 7.v.1967, D.H. Colless [1♀ ANIC; ANIC 33163]; 7 km North North West of Coen, 17.iv.1989, G. & A. Daniels [1♀ AMS; AMK 402266]; Brisbane, C.F. Ashby [1♀ ZFMK]; Dunk Island, 25.viii.1927 [1♀ QM; UQIC 222114]; hut near East Claudie River, Iron Range National Park, 28.xii.1995, G. & A. Daniels [1♀ AMS; AMK 410379]; Eungella National Park, Chelmer'S, Road, 21.132822°S, 148.492683°E, 19.xii.2014, J.H., A.M. & A.W. Skevington [1♂ CNC; CNC384271]; Kuranda, F.P. Dodd [1♂ ANIC, 1♂ 2♀ USNM; ANIC 33166, USNMENT 01028912, 01028876, 01028865]; Leo Creek Roads, McIlwraith Range, 30 km Northeast of Coen, 500 m., 29.vi–4.vii.1976, C.B. & S.R. Monteith [1♀ QM; UQIC 220482]; Mount Glorious, 10.xi.1965, C.F. Ashby [1♀ ANIC; ANIC 33165]; Mount Glorious Scrub Creek Road, Brisbane Forest Park, 17–24.x.1997, N. Power, malaise trap [1♂ QM; UQIC 220481]; Shiptons, Flat, Roberts’ house, 250 m., 1.viii.2004, S. Wright, cleared paddocks [1♀ QM; UQIC 222115]; Shiptons Flat, 15.47°S, 145.14°E, 18.v.1981, D.H. Colless [1♂ USNM; USNMENT 01028856].

#### Description.

Male. Similar to *Allograpta terraenovae* except differs as follows: gena all black; occiput black pilose on dorsal 1/3; scutum shiny except sparsely pollinose on anterior margin, black pilose except yellow pilose on lateral yellow vitta; postalar callus mainly black pilose, with a few intermixed yellow pili anteriorly; calypter brownish black; abdominal fasciae narrower, with a linear posterior margin (not emarginated medially).

*Female*. Similar to male except for normal sexual dimorphism and as follows: frons yellow laterally (about 1/6 of frons width) with a medial, broad, black vitta (about 2/3 of frons width).

#### Distribution.

Australia (Queensland); Fig. 18.

#### Etymology.

The specific epithet is derived from the Latin *notialis* meaning southern (Brown 1956: 731), and it refers to Australia. Species epithet to be treated as an adjective.

## Supplementary Material

XML Treatment for Allograpta alamacula

XML Treatment for Allograpta australensis

XML Treatment for Allograpta javana

XML Treatment for Allograpta terraenovae

XML Treatment for Allograpta notiale
